# Butyrate- and Beta-Hydroxybutyrate-Mediated Effects of Interventions with Pro- and Prebiotics, Fasting, and Caloric Restrictions on Depression: A Systematic Review and Meta-Analysis

**DOI:** 10.3390/life14070787

**Published:** 2024-06-21

**Authors:** Marian Breuling, Elena Tomeva, Nevena Ivanovic, Alexander Haslberger

**Affiliations:** 1 Department of Nutritional Sciences, University of Vienna, A-1090 Vienna, Austria; a11728067@unet.univie.ac.at; 2 HealthBioCare GmbH, A-1090 Vienna, Austria; et@healthbiocare.at; 3 Department of Bromatology, Faculty of Pharmacy, University of Belgrade, 11000 Belgrade, Serbia; nevena.ivanovic@pharmacy.bg.ac.rs

**Keywords:** butyrate, beta-hydroxybutyrate, probiotics, prebiotics, fasting, caloric restriction, depression, major depression disorder

## Abstract

To examine the butyrate- and beta-hydroxybutyrate (BHB)-modulated effects of pre- and probiotic interventions, fasting, and caloric restriction interventions, a systematic literature review was carried out with a subsequent meta-analysis. Three pre-and probiotic intervention randomized control trials (RCTs) were included in the meta-analysis. A significant increase in butyrate (standardized mean difference (SMD) [confidence interval (CI)] 0.34; [0.02–0.67]) and an improvement in depression scores (SMD [CI] 0.15, [−0.35–0.70]) through pre- and probiotic interventions were shown in the meta-analysis. The intervention duration of the included studies ranged from three days to four weeks, with the examined population being healthy adults. Butyrate was measured in either plasma or feces, and the depression score was obtained under the Swedish core affect scale, the hospital anxiety and depression scale (HADS), or the depression, anxiety, and stress scale—21 items (DASS-21). In addition to butyrate, the total SCFA concentration also seems to be positively associated with pre- and probiotic administration (SMD [CI] 0.55 [0.15–0.95]). Despite the significant short-chain fatty acid (SCFA) and butyrate concentration changes, no significant correlation between butyrate and depression or between SCFAs and depression could be shown through linear regression models. Nevertheless, the regression coefficient b1 = 1.57 (*p* = 0.17) for butyrate suggests a strong, positive connection between butyrate and depression. Additionally, three studies were qualitatively analyzed, examining fasting as an intervention and revealing a connection between fasting, BHB, and depression. The association between fasting, BHB, and depression or mood elevation appeared to be related to BHB concentrations, which may be due to the similar biochemical properties of BHB and butyrate. Furthermore, caloric restrictions as alternatives to fasting were proposed as potential long-term interventions.

## 1. Introduction

The scientific interest in the pathology of depression has increased during the last decades due to the rising prevalence of this disorder [1,2,3]. The economic burden of depression in the US increased by about 48% in the last decade, with annual economic damage recently exceeding USD 325 billion per year [4]. The development of depression is influenced by several factors, with family history and genetic predisposition, as well as abuse and stressful events, being strong influencing factors [3,4,5]. In this context, genetic predisposition has been shown to explain about 30% of the variance in the occurrence of major depressive disorder (MDD), with several gene loci already identified, mainly influencing synaptic structures and neuronal transmission, which are associated with this connection [6,7]. Furthermore, epidemiological data show a 2-fold higher major depression disorder (MDD) prevalence in women than in men, strengthening the understanding of MDD as a partially hereditary disease [2].

The high social and economic burden of depression is attributable to the high prevalence in connection with the significant individual burden caused by symptom severity. To describe and understand symptoms more precisely, Phillip W. Gold distinguishes between melancholic and atypical depression. Melancholic depression is thereby defined as the chronic feeling of worthlessness and hopelessness, which manifests in pathological hyperarousal and anxiety, in contrast to atypical depression, which is characterized by a combination of disconnectedness and emptiness [1]. In addition to the high prevalence and the symptoms’ severity, there are also problems regarding depression therapy that are contributing to the high economic, social, and individual burden. In particular, problems with respect to a delayed response to antidepressants, as well as completely treatment-resistant forms of depression, currently comprise the unmet needs of many patients, especially when taking high relapse rates into consideration, which already complicate the treatment of patients with depression [8,9].

Considering these challenges in the pharmacotherapy of depression and the impeded scientific progress due to the still poorly elucidated biological mechanisms of the pathophysiology, it is not surprising that the concept of the gut–microbiota–brain axis has attracted scientific interest in recent years. Indeed, there is growing evidence to support the thesis that the gut microbiota plays an important role in regulating brain function and human behavior and may therefore be involved in the pathophysiology of depression [9,10].

The results from observational studies have suggested that major depression disorder (MDD) leads to an increased occurrence of proinflammatory microbial strains, as well as a decreased relative abundance of short-chain fatty acid (SCFA)-producing bacteria [10]. These findings have strengthened the understanding of the gut and gut microbiota as a depression-modulating entity, with the gut microbiota exerting its influencing potential via different pathways linked to the vagus nerve, the HPA axis, and the enteric nervous system, whereby small molecules such as short-chain fatty acids, in particular, could be crucially involved in the modulating properties of the gut microbiota [11].

SCFAs emerge via the fermentation of dietary fiber through bacteria and can be further subdivided into formate, acetate, propionate, butyrate, valerate, isovalerate, and hexanoate; usually, acetate, propionate, and butyrate are more common because of their occurrence in higher concentrations. This is normally in the ratio of 60:20:20 acetate, propionate, and butyrate [12]. In particular, butyrate production is receiving increasing attention for depression research because butyrate has already shown similar histone deacetylase (HADC) inhibition properties on hippocampal neurons, such as antidepressants [13,14,15]. Butyrate seems to have HADC-inhibiting properties in general, which leads to increased transcription due to the weakened DNA–histone binding complex. Moreover, butyrate can cross the blood–brain barrier (BBB), as it is assisted by butyrate-transporting transmembrane proteins and therefore may be able to influence neurological processes through its modulating properties [15,16].

As short-chain fatty acids (SCFAs) emerge through bacteria, interventions altering microbial compositions, such as pro- and prebiotic administration, are understood as promising approaches that influence SCFA production and concentrations. Although pre- and probiotic administration has shown similar effects, they can clearly be distinguished from each other. Prebiotics are understood as non-digestible but fermentable food components that can be distinguished from dietary fibers by the fact that fibers are metabolized by most colonic microorganisms, while prebiotics are selectively fermented by specific health-promoting bacterial species. Prebiotics are therefore also defined by their health-promoting properties, which are characterized by bacterial strain-specific growth-modulating effects [17,18]. In contrast to prebiotics, probiotics are live microorganisms that also contribute to health when administered. The Food and Agriculture Organization (FAO) defined probiotics as “live microorganisms, which when administered in adequate amounts, confer a health benefit on the host”. With live microorganisms, not exclusively bacterial strains but also health-promoting yeasts are observed and therefore perceived as probiotics [19].

Beta-hydroxybutyrate (BHB) is chemically very similar to butyrate but is not synthesized in the intestine; instead, it is synthesized in the liver as the most important ketone body that provides energy under low-glycemic conditions and especially during periods of fasting or caloric restriction (CR) [17]. Fasting is defined as strongly restricted dietary energy intake with a duration of at least 48 h to reach the optimal medical fasting state, while CR is a reduction in caloric intake without malnutrition and can be applied for prolonged periods [18,19].

BHB can be transported to the brain via the monocarboxylate transporter MCT1, where BHB is an energy substrate for neurons and has a variety of properties that influence brain function. Furthermore, an increase in BHB levels by a ketogenic diet led to increased social behavior in mice, which was modulated via the upregulation of the expression of certain myelin-related genes. In addition, a downregulation of TNFα and Cxcl15 in the hippocampus was found, which all together suggests BHB’s potential to influence neurobiological processes [20]. Since BHB has been associated with similar HADC inhibitory properties as butyrate in in vitro models, similarities emerged in terms of the biochemical influence potential of BHB and butyrate, which should be further investigated by this systematic review and meta-analysis [17].

As shown, a high incidence and a substantial individual burden characterize the sickness of depression disorders, and there is also high public health and healthcare economic relevance. With current evidence suggesting the gut–microbiota–brain axis as influential in the pathophysiology of depression, this paper examines the role of SCFAs, especially butyrate, as influential molecules that mainly modulate the effects of the gut–microbiota–brain axis, with pre- and probiotics being the interventions studied because of their microbial composition and SCFA concentration-altering properties. Due to the chemical similarities of butyrate and BHB, the effects of BHB are also investigated, with fasting and caloric restriction as interventions of interest.

## 2. Materials and Methods

Prisma guidelines for reporting systematic reviews were used (BMJ, https://www.bmj.com/content/372/bmj.n71.short, accessed on 26 May 2024).

### 2.1. Systematic Search

The systematic search was conducted on 11 October 2022, using PubMed and Scopus, as well as Google Scholar for potential gray literature. The advanced search function was used in every database, with the terms being the following: ((((prebiotic* OR postbiotic* OR probiotic* OR synbiotic*) OR (fasting)) OR (“calori* restriction” OR “energy restriction” OR “dietary restriction”)) AND (butyrate OR “beta hydroxybutyrate” OR bhb OR “β hydroxybutyrate” OR “ketone bodies”)) AND (depress* OR “affective disorder” OR” “bipolar disorder”) in PupMed and (prebiotic* OR postbiotic* OR probiotic* OR symbiotic*) OR (fasting) OR (“calori* re- striction” OR “energy restriction” OR “dietary restriction”) AND (butyrate OR “beta hydroxybutyrate” OR bhb OR “β hydroxybutyrate” OR “ketone bodies” AND (de- press* OR “affective disorder” OR “bipolar disorder”) for Scopus. Additionally, Publish or Perish 8 software was used to filter the 500 most fitting search results on Google Scholar that were published between 2000 and 2024. For each of the three search terms, the following were used: (1): (prebiotic*|postbiotic*|probiotic*|synbiotic*) (butyrate|“beta hydroxybutyrate”|bhb|“β hydroxybutyrate”|“ketone bodies”) (depress*|“affective disorder”|“bipolar disorder”); (2): (fasting) (butyrate|“beta hydroxybutyrate”|bhb|“β hydroxybutyrate”|“ketone bod- ies”) (depress*|“affective disorder”|“bipolar disorder”); (3): (“calori* restriction”|“energy restriction”|“dietary restriction”) (butyrate|“beta hydroxybutyrate”|bhb|“β hydroxy- butyrate”|“ketone bodies”) (depress*|“affective disorder”|“bipolar disorder”). The data collection and selection processes were performed by one researcher.

### 2.2. Process of Paper Selection

After the systematic search was conducted, only trials meeting the inclusion criteria were included in the systematic review and meta-analysis. The criteria, therefore, were primary studies and human trials. Although psychiatric illnesses were part of the systematic search, trials examining a population with diagnosed psychiatric illnesses were excluded due to the confounding risk of the parallel administration of specific drugs. Additionally, the administration of potentially influential controls was also an exclusion criterion. To ensure validity, only interventional studies were included in the quantitative analysis. Because no intervention studies were found that analyzed the connection between fasting and depression, other study designs were included, but the connection was therefore only analyzed qualitatively.

### 2.3. Statistical Analysis

Statistical analysis was performed using the software R 4.3.0. For the meta-analysis, the standardized mean difference (SMD) was the effect measure of choice, using change scores, i.e., the differences between post- and pre-intervention. Forest plots were made using the SMD as an effect size estimate, and regression models were produced to calculate the connection between SCFAs, butyrate, and depression. The effect sizes and 95% CI are given. In addition to the overall effect, heterogeneity is also presented in the forest plots. The heterogeneity measure I^2^ provides information about the explainability of the variances of the effect size estimates, with I^2^ = 0% indicating that all effects can be attributed to the interventions. Furthermore, the correlation coefficient b1 is given as the outcome of the conducted regression models, representing the strength of the correlation. The use of the SMD as the effect measure allowed a comparison of different measurement loci and measurement units without the need for additional conversion steps. In two of the three meta-analysis trials included [21,22], only pre- and post-interventional measurements were provided. Consequently, the mean change score was calculated by subtracting pre-interventional values from post-interventional values. The standard deviation (*SD*) of the mean change score was then calculated using the following formula, with the correlation coefficient (*r*) estimated to be 0.5.

$$SD=\sqrt{{SD}_{b}^{2}+{SD}_{f}^{2}-2*r*{SD}_{b}*{SD}_{f}}$$

## 3. Results

**Figure 1:**
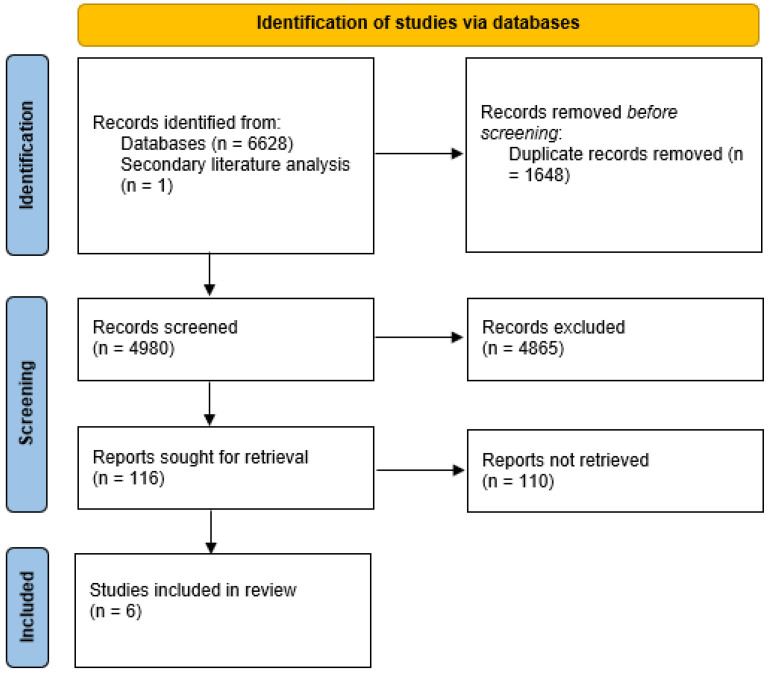
Flowchart illustrating the process of the systematic search.

Like shown in Figure 1, the initial search yielded 122 potential articles from PubMed, 5017 articles from Scopus, and 1500 results in Google Scholar. Altogether, 6628 articles were collected, of which 1648 were recorded more than once and deleted using the Rayyan program. In total, 4980 individual studies remained, and 1 additional study was found during the secondary literature analysis, leaving 4981 studies available for the title and abstract screening process. Then, 4865 articles were excluded because they did not meet the inclusion criteria, resulting in 116 studies that were suitable for full-text screening. Finally, six studies were eligible for the systematic review, of which three were RCTs that examined the administration of pro- and prebiotics, and they were used to carry out a meta-analysis [23,24,25] (Table 1), while three observational studies investigating the characteristics of fasting and caloric restrictions were included in a qualitative analysis [21,22,26] (Table 2).

**Figure 2:**
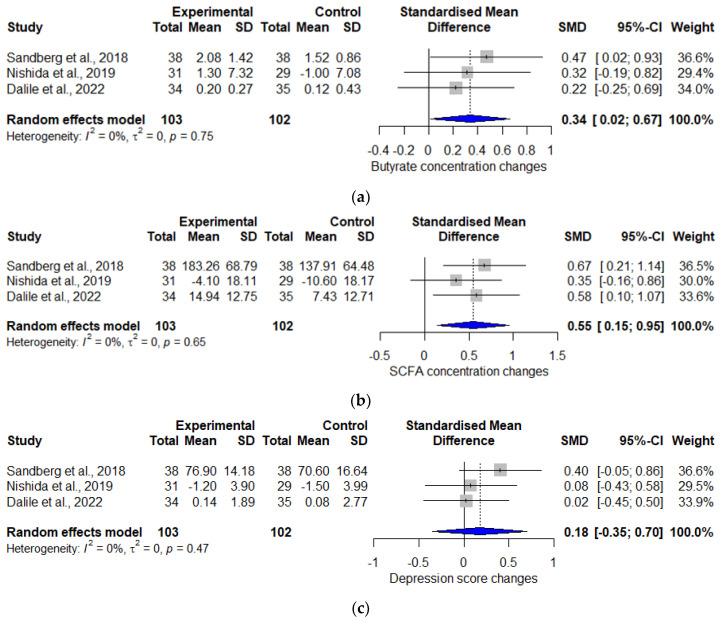
Forest plots illustrating the SMDs of butyrate (**a**), total SCFAs (**b**), and depression score changes (**c**) [23,24,25].

The overall effect of the interventions on butyrate, SCFAs, and depression scores are shown in Figure 2a–c, respectively. Several forest plots are presented, evaluating and illustrating the overall effect of interventions on butyrate (Figure 2a), SCFAs (Figure 2b), and depression scores (Figure 2c).

Interventions with pro-and prebiotics led to significant butyrate and SCFA concentration changes, with an SMD [CI] of 0.34 [0.02–0.67] for butyrate (Figure 2a) and 0.55 [0.15–0.95] for SCFAs (Figure 2b). The overall SMD of the depression score changes was not significant at 0.18 [−0.35–0.7] but favored the interventions (Figure 2c).

In order to determine a possible correlation between butyrate or SCFAs and the change in depression scores, a regression model was calculated. No significant correlation could be shown for butyrate (*p*-value = 0.17) and SCFAs (*p*-value = 0.44), but the correlation coefficient b1 was positive in both models ((b1 = 0.75; SCFA) and (b1 = 1.57; butyrate)). This not only suggests that butyrate is able to ameliorate depression but also that increasing propionate, acetate, valerate, and hexanoate levels might limit butyrate’s depression-ameliorating properties.

## 4. Discussion

Previously conducted trials showed a promising connection between pro- and prebiotic administration and depression and/or anxiety. Liu et al. showed—in a systematic review and meta-analysis with 36 controlled trials included—that probiotic interventions decreased depression severity across the studies, with the effects seeming to be strain- and administration duration-specific, while no significant connection between prebiotic administration and depression was found [27]. Similar effects were shown in a meta-analysis carried out by Huang et al., in which five trials were included, and Hofmeister et al. also observed the same results in 44 included trials [28,29]. This evidence suggests a connection between pro- and prebiotic administration and depression, and the modulation pathway needs to be further examined.

Moreover, this conducted meta-analysis of the three RCTs (Table 1) showed a significant increase in SCFAs and especially butyrate through probiotic and prebiotic administration. These findings are further supported by the other literature examining pro- and prebiotic administrations. Multiple recent studies were able to show that prebiotics increase SCFAs and butyrate production [30,31,32,33]. The two prebiotic studies included in the meta-analysis used extruded wheat bran and rye-based bread as prebiotic interventions. These consisted mainly of indigestible starch, which is suitable as a reactant for butyrate formation, as shown by the positive effects on butyrate and SCFA production in the included prebiotic intervention studies. In the probiotic intervention study, Lactobacillus gasseri was supplemented as a probiotic, which has already been shown to increase SCFA concentrations in interventional studies [34,35].

Because not many trials investigated the effect of pro- and prebiotic administration on depression and SCFAs, all trials corresponding to the inclusion criteria also were included in the meta-analysis, although SCFA measurement loci or depression detection was different. Values were therefore normalized by calculating the SMD. This was carried out by dividing the difference of the mean outcomes between the control group and the intervention group through the SD of the outcome among participants [36], resulting in scale-independent values.

Interestingly, inter-individual variations affect prebiotic- and probiotic-induced butyrate production, altering the overall butyrate concentration [37]. Holmes et al. demonstrated differences in the composition of the microbiota of high butyrate producers compared to low butyrate producers, highlighting the dependence of bacterial composition on SCFA and butyrate formation. Firmicutes species, especially Lachnospiraceae, have been identified as the main producers of SCFAs and contribute considerably to butyrate production, whereas Bacteroidetes appear to have no butyrate-forming properties [37]. These results also suggest that the efficacy of pre- and probiotic interventions depends on the bacterial composition [38]. Therefore, a question arises: which conditions favor prebiotic and probiotic administration? It is plausible that the effect of probiotics is better under dysbiotic conditions, as the modulation of the composition of intestinal bacteria has long-lasting effects, whereas the success of prebiotic administration seems to be more promising when the intestinal microbiome is well composed. This is due to different mechanisms for increasing butyrate concentration. Probiotics increase the concentration of butyrate-producing bacteria, while prebiotics provide substrates for the formation of SCFAs and therefore already require strains of butyrate-producing bacteria.

Although no significant correlation was found between depression and butyrate in the linear regression model, a clear trend suggests that the properties of butyrate have great potential for brain health and improvement in depression. Interestingly, the correlation between butyrate and depression (b1 = 1.57) is stronger than that between SCFAs and depression (b1 = 0.75), supporting the hypothesis that butyrate mainly mediates the effects of SCFAs on depression. Interestingly, propionate was negatively associated with depression, although no statistical significance was reached [b1 = −0.61 (*p*-value = 0.57)]. This indicates that other SCFAs, especially propionate, could inhibit the butyrate-mediated effects on depression.

Because energy reduction leads to increased ketone body and BHB production, fasting and caloric restriction are the most appropriate interventions to investigate the potential of BHB to alleviate depression symptoms and improve mood. Both ketone body and BHB concentrations were significantly increased by fasting, whereas no matching studies were found that examined caloric restriction as an intervention. Usually, CR results in an energy reduction of about 10 to 25% which also results in a significant increase in BHB concentration, as shown in animal and human studies [39]. Fasting and CR are both methods of energy reduction that are associated with weight loss, life-prolonging, and anticancer properties [39]. Although both methods have similar health effects, there are several notable differences regarding BHB production and, subsequently, their impact on depression.

Since Toledo et al. (Table 2) found a plateau of BHB concentration reached after 5 days of fasting, as well as a rapid reduction in BHB levels after the end of the fasting period, no persistent BHB increase seems to be possible through fasting interventions. Furthermore, significantly enhanced emotional wellbeing was shown through the fasting intervention [26].

Castro et al. (Table 2) found a similar plateau effect of ketone body production through the very low-calorie diet (VLKD). Moreover, quality of life (QoL) was assessed as the variable representing depression and increased from measurement point to measurement point under the fasting regime [21].

In a study by Yang et al. (Table 2) that examined the effects of ten days of ad libitum water fasting on depression score changes and ketone body concentrations, a similar ketone body concentration plateau seemed to be reached after day 5, with stable concentration measurements until the end of the fasting intervention. Psychological parameters all behaved similarly and dropped during the first days of fasting to finally reach baseline values after the interventional duration. Interestingly, the decreasing psychological parameter trend ended on day 3 for depression dejection and day 6 for self-rated anxiety, suggesting that high ketone body concentrations may alleviate fasting-induced psychological impairments [22].

Because CR is less severe and, therefore, can be implemented for months or years, continuous high BHB levels are just possible with CR, suggesting CR’s ability to generate longer-lasting effects. Nevertheless, these effects are strongly limited by the participants’ compliance relative to energy reduction interventions, which is often low because of the high drop-out rates due to hunger and cravings [40].

In summary, in the qualitative analysis, a significant improvement with respect to depression through fasting was found in two of the three studies (Table 2), except for water and ad libitum fasting. This suggests that the severity of the fast correlates strongly with depression parameters and that fasting from 200 to 800 kilocalories per day correlates positively with improvements in depression scores.

In analyzing improvements in depression, it is important to consider that even if BHB levels were increased by the intervention, the effects on depression may not be related to BHB, because in an overweight or obese population, weight loss modulated by fasting could also be responsible for improved wellbeing. This needs to be investigated in further studies examining the effects of fasting and caloric restrictions on depression in healthy, normal-weight adults.

Taken together, there is strong evidence that butyrate and BHB have positive effects on depression. Nevertheless, further studies on the pathophysiology of depression disorder and the molecular mechanisms of SCFAs are needed.

Recent studies suggest that MDD is a neurogenerative disorder due to insufficient levels of neurotrophic factors, particularly the brain-derived neurotrophic factor (BDNF), in certain brain regions. Studies in mice have shown that BHB can activate the cAMP-response element binding protein (CREB) and nuclear factor κ-B, a DNA transcription; cytokine production; and the cell survival protein complex. Furthermore, BDNF expression in neurons was enhanced through BHB, suggesting the HDAC inhibition of genes associated with BDNF to be one key mechanism of action that not only balances the reduced BDNF levels of MDD patients but also improves the immune system and reduces inflammation as further depression-improving mechanisms [41]. The neuroinflammation-reducing properties of BHB may be further mediated through protein kinase B (Akt), the inhibition of which abolished BHB’s positive influence on microglial polarization, phagocytosis, and inflammation [42]. In contrast to BHB, the transcriptional regulatory properties of which have only recently been discussed, butyrate was one of the first endogenous substances known to inhibit HADC activity. From this observation, the link between butyrate and depression emerged, as BDNF genes in the prefrontal cortex showed increased acetylation induced by butyrate [16]. Moreover, butyrate seems to increase glutathione (GSH) production by increasing enzyme expression. This could be another relevant mechanism of action due to the large antioxidant capacity of GSH and its potential to reduce neurodegenerative risk factors such as hydrogen peroxides and lipid peroxides [43].

BDNF appears to be involved in each case, but whether there is a difference in the modulation of BDNF levels by butyrate or BHB remains to be investigated for the successful development of new drugs and the optimal administration of pre- or probiotics or energy restriction therapies in terms of disease specificity.

## 5. Limitations of This Study

The limited inclusion of studies in our systematic review was observed due to a scarcity of trials examining the relationship between pre- and probiotic administration, fasting, CR, and mental health alongside SCFA changes. Furthermore, methodological inconsistencies in SCFA measurement methods, encompassing both fecal and plasma analyses, posed challenges for data synthesis. Despite these limitations, this review provides valuable insights into the potential effects of these interventions on mental health outcomes.

## 6. Conclusions

Although several brain disorders are already associated with dysbiotic conditions, questions regarding the characteristics of the connection are not sufficiently answered yet. In particular, with respect to the actuator of changes, whether alterations in microbial compositions are induced by brain signaling or whether brain dysfunction is driven by changes in the gut microbiota is not known [12]. As shown in this review, butyrate appears to be involved in the link between the microbiota–brain axis and depression, although the underlying mechanisms are not yet fully understood. Considering the positive effects of BHB on brain health and depression and the similarities of butyrate and BHB in terms of their HDAC inhibition potential, the potential of butyrate and BHB to alter transcriptional processes appears to be a key mechanism of the link between butyrate, BHB, and depression.

The interventions studied showed BHB- and butyrate-inducing properties, suggesting a positive potential for depression therapy through pro-and prebiotic administration, fasting, and caloric restriction. However, further studies are needed to clarify which parameters influence the efficacy and strength of the connections in order to determine the best-performing dietary regimes or pro- and prebiotic interventions and select them as depression-preventive or therapeutic options in the future.

## Figures and Tables

**Table 1:** Pre- and probiotic RCTs included in the meta-analysis.

Study	Country	Population	Age (SD)	Sex (% Female)	Study Type	Intervention	Control	Duration	Outcomes
Sandberg et al., 2018, [23]	Sweden	38 healthy subjects (52–70 years)	63.6 (5.3)	78.9	RCT; crossover design	Rye-based bread; 75 g of carbohydrates daily; 5.7 g of soluble fiber	White wheat bread; 75 g of carbohydrates daily; 1.2 g of soluble fiber	3 days	Physiological: Plasma SCFAs Psychological: Swedish core affect scale
Nishida et al., 2019, [24]	Japan	60 healthy, young adults	25.1 (0.6)	31.6	RCT; placebo, parallel group	Lactobacillus gas- seri 1 × 10^10^ cfu	placebo	24 weeks	Physiological: Fecal SCFAs Psychological: HADS
Dalile et al., 2022, [25]	Belgium	69 healthy, men (20–40 years)	26 (4.1)	0	RCT; placebo, parallel group	Extrusion of cooked wheat bran; 25 g of fiber per day	MCC cereal as a placebo; 25 g of hardly fermentable fiber	4 weeks	Physiological: Fecal SCFAs Psychological: DASS-21

Abbreviations: Standard deviation (SD); randomized controlled trail (RCT); microcrystalline cellulose (MCC); short-chain fatty acids (SCFAs); hospital anxiety and depression scale (HADS); depression, anxiety, and stress scale—21 items (DASS-21).

**Table 2:** The qualitative review included observational trails, investigating the effects of fasting interventions on depression.

Study	Country	Population	Age (SD)/ BMI (SD)	Sex (% Female)	Study Type	Intervention	Duration (SD)	Outcomes	Findings
Toledo et al., 2019, [26]	Germany	1422 subjects (18–99 years)	55.4 (0.4)/ 28.2 (0.2)	59.1	Observational study	200–250 kcal per day with 25–35 g carbohydrates	Mean 8.2 (0.1)	Physiological: acetoacetic Psychological: emotional wellbeing (EWB)	↑ Acetoace- tic * ↑ EWB * EWB effects increased with fasting length *
Castro et al., 2018, [21]	Spain	20 subjects (18–65 years; BMI ≥ 30 kg/m^2^)	47.2 (10.2)/ 35.5 (4.4)	60.0	Observational study	VLKD (from weight loss program); 600–800 kcal/day until targeted weight loss is reached	Depends on weight loss progress and targeted weight	Physiological: BMI, BHB Psychological: QoL	↑ BHB * ↑ QoL *
Yang et al., 2021, [22]	China	13 (28–55 years)	39.6 (8.1)/ n.a.	0	Observational study	Water-only fasting	10 days	Physiological: Ketone bodies Psychological: POMS, SDS	↑ Ketone bodies * ↓ SDS score *

Positive developments are marked with ↑; Negative developments are marked in ↓; Significant changes (*p* ≤ 0.05) are marked with *. Abbreviations: Body mass index (BMI); standard deviation (SD); very low-calorie diet (VLKD); quality of life (QoL); profile of mood states (POMS); self-rating depression scale (SDS).

## Data Availability

All created data are mentioned in this review.
